# Quality of life after acute myocardial infarction: A comparison of diabetic versus non-diabetic acute myocardial infarction patients in Quebec acute care hospitals

**DOI:** 10.1186/1477-7525-3-80

**Published:** 2005-12-05

**Authors:** Ewurabena Simpson, Louise Pilote

**Affiliations:** 1 Division of Clinical Epidemiology, the Montreal General Hospital Research Institute, Montreal, Quebec, Canada

## Abstract

**Background:**

Previous studies have evaluated the individual effects of acute myocardial infarction (AMI) and diabetes mellitus on health-related quality of life outcomes (QOL). Due to the rising incidence of these comorbid conditions, it is important to examine the synergistic impact of diabetes mellitus and AMI on QOL.

**Methods:**

In this study, we assessed using several previously validated questionnaires the QOL and functional status of 96 diabetic patients and 491 non-diabetic patients admitted to Quebec hospital sites with AMI between 1997 and 1998. We also conducted multivariate analyses to ascertain whether diabetes mellitus was an independent determinant of SF-36 physical functioning (PCS) and mental health (MCS) component score QOL outcomes after AMI.

**Results:**

Both patient groups had similar baseline clinical characteristics, but diabetic patients had slightly higher rates of cardiac risk factors compared to non-diabetics. Overall, QOL measures were similar between both patient groups at baseline, but diabetic patients reported poorer functional status than non-diabetic patients. Over the study period, there were significant differences between the QOL and functional status of diabetic and non-diabetic populations. By one year, diabetic patients reported poorer QOL outcomes than non-diabetic patients. However, diabetic patients showed greater improvements in their functional status, but were less likely to return to work compared to non-diabetic patients. In contrast with these findings, our multivariate analyses showed that diabetes mellitus was not an independent determinant of QOL and functional status.

**Conclusion:**

Our study findings suggest that diabetes mellitus is not an independent determinant of QOL after AMI.

## Background

Several clinical studies have shown that acute myocardial infarction (AMI) causes a decline in the social, physical and psychological functionality of affected patients [1-12]. These changes in quality of life (QOL) can impair the patient's ability to perform even basic daily tasks. Similarly, various studies have found that diabetes mellitus is also associated with poorer QOL. Both type 1 and type 2 diabetes mellitus have been associated with negative socioeconomic changes, increased morbidity, worsened physical capacity and overall declines in general health status [13-18]. Because diabetes mellitus is so closely associated with coronary artery disease, it is important to evaluate the synergistic effect of these conditions on QOL following AMI. Clinicians will be able to use this information to establish appropriate health management strategies for patients who suffer from both of these diseases.

The purpose of this paper is to measure and compare QOL outcomes for diabetic and non-diabetic patients who have sustained a Q-wave or non Q-wave AMI. Specifically, this paper aims to address 1) whether diabetes mellitus influences the QOL of post-AMI patients and 2) which QOL dimensions are most or least affected by the diagnosis of diabetes mellitus.

## Methods

### Patient cohort & QOL measurement

From January 1997 to November 1998, 587 Quebec patients with a confirmed Q wave or non-Q wave AMI were enrolled in a 1-year prospective cohort study of QOL after AMI, as detailed previously [19]. Patients who were eligible for the study were admitted to one of 10 Quebec hospital sites, were able to read and understand French or English, and had survived at least 24 hours after hospital admission. We excluded patients if they were not capable of giving informed consent or responding to a questionnaire. Diabetic status and additional baseline demographic and clinical characteristics were determined at the time of enrollment by a study nurse. A patient was classified as diabetic based on a description of diabetic status in chart notes, regular use of antihyperglycemic medications, and/or laboratory values for Hemoglobin A_1_C.

We measured changes in patient QOL by means of questionnaires completed by the patients at baseline admission, at 30 days, at 6 months, and at 1 year following AMI. We relied on previously validated questionnaires to assess the patients' overall health perception, namely the SF-36 health survey [20], a visual analogue scale to rate overall QOL (range from 0, poorest QOL to 100, best QOL) that was adopted from Torrance's Feeling Thermometer [21,22] and the EuroQol measure [23], and a five-level scale obtained from the National Health Interview Survey [24]. To measure the patients' functional status, we used the Duke Activity Status Index (DASI) and a single four-level question to compare overall functioning before and after AMI [25]. In addition, each patient reported his or her level of optimism using another four-level scale to rate expectations of returning to a normal lifestyle [26]. We also measured patients' work status and their ability to return to work using an instrument developed for the Study of Economics and Quality of Life [27]. As a final measure of physical and mental functioning, we created a physical component summary score (PCS) and a mental component summary score (MCS) as described by Ware et al [20] by combining the physical components (physical functioning, role limitations due to physical problems, bodily pain and vitality) and the mental components (social functioning, role limitations due to emotional problems, mental health and general health perceptions) of the SF-36 subscales.

First, we conducted a univariate analysis to compare the raw outcomes for diabetic versus non-diabetic patients. For SF36 scores, differences of 5 points were considered clinically significant. P-values of <0.05 were considered statistically significant. A multivariate linear regression model was then created to obtain adjusted comparisons of the QOL scores for physical and mental health, and to isolate any demographic, clinical, and psychosocial baseline characteristics that influenced patient QOL 1 year after AMI. Variables that were included in the multivariate model were: baseline score, diabetes, sex, age, education, congestive heart failure, previous coronary artery bypass surgery (CABG), previous percutaneous transluminal coronary angioplasty (PTCA), ventricular fibrillation, recurrent ischemia, previous angina, and hypercholesterolemia. An optimal model was estimated using backward and forward model selection algorithms that have been previously described [28].

## Results

### Baseline characteristics

Of the 587 enrolled patients, we identified 96 (16%) diabetic patients and 491 (84%) non-diabetic patients. In general, the diabetic and non-diabetic patients had similar demographic and clinical characteristics at baseline, but there were some clinically significant differences between the groups (Table 1). At baseline, there was a higher proportion of women in the diabetic population compared to the non-diabetic population (33% versus 19%). Diabetic patients also tended to be older than the non-diabetic patients at enrollment (66 years and 60 years, respectively). In terms of cardiac risk factors, the diabetic patients had higher rates of angina (34% versus 23%), previous AMI (27% versus 20%), and hypertension (60% versus 31%) when compared to non-diabetic patients. Similarly, there were more diabetic patients who had experienced an AMI of Killip class I or greater (29% versus 16%, for diabetics and non-diabetics).

**Table 1:** Demographic and clinical characteristics of diabetic patients and non-diabetic patients at baseline hospitalization

	**Diabetic N = 96**	**Non-diabetic N = 491**
**Demographic characteristics**		
Males	67	81
Mean age (years)	66	60
Caucasian race	92	96
Married	68	71
Education (mean years)	10	11
Length of stay (mean days)	10	8
**Clinical History**		
AMI	27	20
Angioplasty	7	6
Bypass surgery	6	7
**Cardiac risk factors**		
Hypertension	60	31
Current smoking	30	42
Hypercholesterolemia	34	37
**Characteristics of AMI**		
Anterior location	36	32
Inferior location	38	43
Lateral location	22	20
Q wave	49	49

Values are given as percentages of n unless otherwise indicated.

For in-hospital procedures received at baseline, there were several clinically significant differences in the use of revascularization procedures within the two patient populations (Table 2). Fewer diabetic patients were initially hospitalized at sites with angiography availability (50% versus 58%). During baseline hospitalization, fewer diabetic patients underwent coronary angioplasty than non-diabetics regardless of whether or not they were hospitalized at sites with angiography availability (15% versus 24%). Diabetic patients were also less often treated with coronary angioplasty even after undergoing angiography (36% versus 56%).

**Table 2:** Use of cardiac procedures for diabetic patients and non-diabetic patients during baseline hospitalization

	**Diabetic N = 96**	**Non-diabetic N = 491**
**Procedures at baseline**		
Angiography	41	43
Angioplasty	15	24
Bypass surgery	14	7
Revascularization	27	30
Time to angiography (median days)	5 (3,10)	6 (2,10)
**Characteristics of angiography**		
**Diseased coronary vessels**		
None	3	7
One	26	45
Two	33	25
Three	36	20
Left main	10	11
Left ventricular ejection fraction	40 (35,50)	50 (35,60)
**Procedure following angiography**		
Angioplasty	36	56
Bypass surgery	31	15

Values are given as percentages of n except for continuous variables for which the inter-quartile ranges are given in parentheses.

### Quality of life and medical outcomes

#### Overall health perception

We obtained complete follow-up QOL measures for over 80% of the study patients (Table 3). In general, diabetic patients reported lower QOL outcomes than the non-diabetic patients for all SF-36 domains at baseline, as well as after 1 year of follow-up (Table 3). However, when we analyzed the mean differences between these scores, the majority of these differences were not clinically significant (Table 3). Physical functioning was the only dimension where there was a clinically significant difference, and diabetic patients had average scores that were -14.3 points worse than those for the non-diabetic patients (95% confidence interval [CI] -20.7,-7.8).

**Table 3:** Mean SF-36 score differences between diabetic patients and non-diabetic patients

	**Baseline N = 587**	**6 months N = 480**	**1 year N = 491**
Physical functioning	-16.7 (-23.1,-10.3)	-13.6 (-21.0,-6.2)	-14.3 (-20.7,-7.8)
Role-physical	-13.1 (-22.7,-3.5)	-10.3 (-20.7,0.6)	-14.0 (-25.9,-3.2)
Bodily pain	-7.2 (-13.5,-0.9)	-5.2 (-12.2,1.7)	-4.9 (-11.3,1.5)
General health	-11.8 (-16.8,-6.7)	-7.2 (-12.9,-1.5)	-9.3 (-14.9,-3.6)
Vitality	-2.5 (-7.5,2.6)	-6.0 (-11.7,-0.4)	-2.7 (-7.9,2.5)
Social functioning	-7.3 (-13.1,-1.6)	-7.7 (-14.9,-0.5)	-6.4 (-12.3,-0.4)
Role-emotional	-4.9 (-14.4,4.6)	-8.8 (-19.6,-2.1)	-6.1 (-16.6,4.4)
Mental health	0.6 (-4.1,5.3)	-0.4 (-5.2,4.4)	-2.5 (-8.1,3.1)
Physical component summary (PCS)	-6.0 (-8.4,-3.6)	-4.6 (-7.5,-1.7)	-5.3 (-7.9,-2.7)
Mental component summary (MCS)	0.9 (-1.7,3.5)	-1.0 (-3.8,1.8)	-0.3 (-3.2,2.6)

Differences are given as the diabetic patient scores minus the non-diabetic patient scores with the 95% confidence intervals in parentheses.

Differences are considered to be clinically significant when the confidence interval laid ±5 units from zero.

Results from the Torrance/EuroQOL Health Perception Scale indicated that, on average, both diabetic and non-diabetic patients saw improvements in their overall health after 1-year of follow-up (Table 4). Nonetheless, the scores for the diabetic patients were significantly lower scores than those for the non-diabetic patients at 1 year (mean difference of -8.7 (95% CI -12.7,-4.6)).

**Table 4:** Changes in quality of life for diabetic patients versus non-diabetic patients

	**Diabetic**		**Non-diabetic**	
	**Baseline N = 96**	**1 year N = 73**	**Baseline N = 491**	**1 year N = 418**

**Health Perception Scale **(mean score)	65.1 (60.6,69.5)	65.3 (61.1,69.5)	70.8 (68.9,72.6)	74.0 (72.3,75.6)
**Duke activities status index **(mean score)	19.4 (16.4,22.3)	20.8 (17.6,23.9)	30.0 (28.3,31.6)	29.9 (28.2,31.6)
**General health perception**				
Good – Excellent	56.2	73.6	67.6	82.5
Fair – Poor	44.8	27.4	32.4	17.5
**Abilities to perform tasks**				
Can do anything/almost anything	56.4	50.7	72.3	70.5
Trouble with some things/anything	45.6	49.3	27.7	29.5
**Optimistic about returning to normal health**				
Strongly agree/Agree	71.7	61.7	75.4	67.9
Disagree/Strongly disagree	5.4	15.0	6.1	10.8
**Work status**				
Full-or part-time work	34.1	14.3	53.3	36.1
Sick leave	2.2	1.4	1.7	3.0
Other	63.7	82.3	45.0	59.0

Values are given as percentages of n unless otherwise indicated.

**Figure 1:**
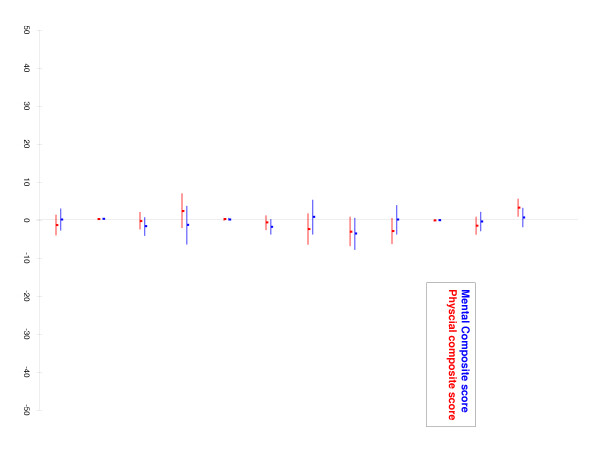
Adjusted mean SF-36 score differences between diabetic patients and non-diabetic patients at 1 year.

Despite these raw differences in QOL outcomes, multivariate analyses for the physical functioning composite score (PCS) and the mental health composite score (MCS) at 1 year showed that, after adjustment for baseline prognostic factors, a diagnosis of diabetes mellitus was not associated with poorer QOL after AMI (Figure 1). Our models showed that higher baseline SF-36 scores were associated with higher PCS and MCS results at 1 year (β-coefficients of 0.39 (95% CI 0.30, 0.48) and 0.42 (95% CI 0.33, 0.50), respectively). Level of education and male sex were also associated with higher PCS scores at 1 year follow-up (β-coefficients of 0.26 (95% CI 0.06, 0.47) and 3.3 (95% CI 1.1, 5.6), respectively). Increased patient age was associated with lower PCS results at 1-year (β-coefficient of: -0.08 (95% CI -0.16, 0.01)). Thus, our multivariate models suggest that differences in QOL scores at 1 year between diabetic and non-diabetic patients were confounded by the lower baseline QOL scores, lower level of education, higher proportion of women, and increased age of the diabetic population.

#### Functional status

Overall, both patient groups showed modest improvements in their mean DASI outcomes from baseline to 1-year follow-up (Table 4). At baseline, the mean DASI score for the non-diabetic group was significantly better than that of the diabetic group (mean difference of -10.6 (95% CI -14.0, -7.2)). After 1 year of follow-up, both groups showed modest improvements in their DASI outcomes and the mean difference decreased to -9.1 (95% CI -12.3, -5.9). Despite improvements in their DASI scores, diabetic patients reported poorer functioning than non-diabetic patients at 1 year following the AMI (51% versus 71% said that they can do anything/almost anything).

For the assessment of patient optimism 1 year after AMI, there were fewer diabetic patients than non-diabetic patients who reported optimism about returning to a normal lifestyle after their AMI (62% versus 68%, respectively). However, both groups showed declines in their levels of optimism from baseline to 1 year (change of -10 and -8 for diabetics and non-diabetics, respectively).

#### Work status

For employment status at baseline and at 1 year, fewer diabetic patients were engaged in full or part-time employment (14% versus 36% non-diabetics at 1 year). Multivariate analysis indicated that, when all prognostic factors included in the SF-36 models were considered, the number of diabetics who were employed was similar to the number of employed non-diabetics.

## Discussion

The results of our linear regression models suggest that there is no clinical or statistical significant difference between the QOL of diabetic and non-diabetic patients after AMI. Although the diabetic patients reported lower QOL results than non-diabetics 1-year after AMI, our regression models for physical functioning and mental health composite scores showed that these differences could be attributed to the diabetics' poorer QOL characteristics at baseline. Furthermore, the differences between QOL scores for the two patient groups were also confounded by the increased age, higher proportion of women and lower levels of education of the diabetic patient population.

In general, our diabetic patients had more severe disease than the non-diabetic patients at baseline. At baseline, the diabetic patients had more cardiac risk factors (Table 1) and more extensive coronary artery disease (Table 2) relative to the non-diabetic patients. Moreover, diabetic patients were hospitalized for more days than non-diabetic patients and had more severe AMI events than the non-diabetic patients, which suggests that diabetic patients had more complicated hospital courses than the non-diabetic patients (Table 1). Of the patients who underwent cardiac angiography, diabetic patients showed a higher number of diseased coronary vessels than non-diabetics (Table 2). Previous studies have shown clinical characteristics of coronary artery disease are important determinants of morbidity and mortality after an initial AMI. However, our regression model did not show any significant correlations between these clinical characteristics and patient QOL 1 year after AMI (Figure 1).

In our study, diabetic patients received fewer invasive procedures than non-diabetic patients did following an AMI event (Table 2). These results are in line with previous findings which suggest that diabetic patients do not receive optimal secondary prevention procedures and medications after an AMI [29]. From our study, it is difficult to conclude whether these trends in cardiac procedures had an effect on the patients' QOL. Up to now, there have been conflicting data about the effects of invasive cardiac procedures on QOL after myocardial infarction. More recent data from the same authors indicate that cardiac procedures do not significantly affect QOL 1 year after AMI [19].

Although it was not assessed in this study, the diabetic patients' slower rate of return to work may have been associated with differences in their baseline demographic and clinical characteristics. For example, in the older diabetic population, it is possible that more of the patient had already reached or were close to the normal age of retirement when their AMI occurred, which would have influenced their decision to return to work. As discussed earlier, the diabetic patients also tended to have more severe coronary artery disease characteristics and associated morbidity than the non-diabetic patients (Tables 1 and 2). These poorer clinical characteristics were likely confounders that influenced the rate return to work for the diabetic patient group.

There were several limitations to this study. First, the size of the diabetic patient group was not very large because the patients were not recruited based on their diabetic status, when the original study was designed. As a result, our sample sizes are more representative of prevalence of diabetes mellitus among patients with ischemic heart disease. Our sample size was further limited as there were more diabetic patients than non-diabetic patients who were lost to follow-up over the study period (27% versus 14%, respectively).

Other limitations to this study were the various demographic differences between the two patient groups at baseline. These differences were accounted for as much as possible in our linear regression model, but it is possible that we did not include other all the contributory variables in our model.

## Conclusion

Our study findings suggest that a diagnosis of diabetes mellitus is not an independent determinant of QOL after AMI. Similar to the non-diabetic patients, the diabetic patients showed correlations between their QOL and their baseline scores, age, sex, and level of education.

## Authors' contributions

All authors have made substantial contributions to conception and design, or acquisition of data, or analysis and interpretation of data. They have been involved in drafting the article or revising it critically for important intellectual content and they have given final approval of the version to be published. Both authors have participated sufficiently in the work to take public responsibility for appropriate portions of the content. They have read and approved the final manuscript.
